# Lysine Acetylation Reshapes the Downstream Signaling Landscape of Vav1 in Lymphocytes

**DOI:** 10.3390/cells9030609

**Published:** 2020-03-04

**Authors:** Sonia Rodríguez-Fdez, Lucía Fernández-Nevado, L. Francisco Lorenzo-Martín, Xosé R. Bustelo

**Affiliations:** 1 Centro de Investigación del Cáncer, CSIC-University of Salamanca, 37007 Salamanca, Spain; soniarf@usal.es (S.R.-F.); lnevado@usal.es (L.F.-N.); Fran_lm@usal.es (L.F.L.-M.); 2 Instituto de Biología Molecular y Celular del Cáncer, CSIC-University of Salamanca, 37007 Salamanca, Spain; 3 Centro de Investigación Biomédica en Red de Cáncer (CIBERONC), CSIC-University of Salamanca, 37007 Salamanca, Spain

**Keywords:** Vav, guanosine nucleotide exchange factor, Rac1, Rho, JNK, NFAT, T cell receptor, acetylation, tyrosine phosphorylation, adaptor

## Abstract

Vav1 works both as a catalytic Rho GTPase activator and an adaptor molecule. These functions, which are critical for T cell development and antigenic responses, are tyrosine phosphorylation-dependent. However, it is not known whether other posttranslational modifications can contribute to the regulation of the biological activity of this protein. Here, we show that Vav1 becomes acetylated on lysine residues in a stimulation- and SH2 domain-dependent manner. Using a collection of both acetylation- and deacetylation-mimicking mutants, we show that the acetylation of four lysine residues (Lys^222^, Lys^252^, Lys^587^, and Lys^716^) leads to the downmodulation of the adaptor function of Vav1 that triggers the stimulation of the nuclear factor of activated T cells (NFAT). These sites belong to two functional subclasses according to mechanistic criteria. We have also unveiled additional acetylation sites potentially involved in either the stimulation (Lys^782^) or the downmodulation (Lys^335^, Lys^374^) of specific Vav1-dependent downstream responses. Collectively, these results indicate that Nε-lysine acetylation can play variegated roles in the regulation of Vav1 signaling. Unlike the case of the tyrosine phosphorylation step, this new regulatory layer is not conserved in other Vav family paralogs.

## 1. Introduction

Nε-lysine acetylation is a posttranslational modification that was discovered on histones in 1964 [1,2,3]. However, unlike the case of phosphorylation steps, the relevance of acetylation in the regulation of protein function was not fully appreciated until recent times due to the lack of proper detection reagents. This situation changed at the beginning of this century upon the development of both high-resolution mass spectrometry techniques and acetyl lysine-specific antibodies. These advances led to the identification of a wide range of regulatory functions for lysine acetylation in a large variety of nuclear, cytosolic, plasma membrane, and organelle resident proteins [2,3]. Unlike the case of phosphorylation, the stoichiometry of protein acetylation is usually low [2,3], suggesting that it might affect either specific pools of the target proteins or a very limited time window of the functional cycle of the proteins that become acetylated. Due to this, the actual relevance and time of action of this posttranslational modification remains as yet unknown for most acetylated proteins.

The Vav family is a group of signal transduction proteins that work both as guanosine nucleotide exchange factors (GEFs) for Rho GTPases and adaptor molecules. This family has single representatives in invertebrates and three members in most vertebrate species (Vav1, Vav2, and Vav3) [4,5]. These proteins play critical functions in lymphopoiesis, osteogenesis, cardiovascular homeostasis, neuronal-related processes, and nematode tissue rhythmic behaviors [4,5,6,7,8,9,10,11,12,13,14,15,16]. They are also involved in human diseases such as cancer, multiple sclerosis, immune-related deficiencies, and the life cycle of a number of pathogens inside mammalian cells [4,5,17]. Vav1 is the family member that displays a more restricted expression, since it is preferentially detected in hematopoietic cells [4,5]. In line with this, its evolutionary origin parallels the development of the adaptive immune system that took place at the level of jawed fish [5].

**Figure 1:**
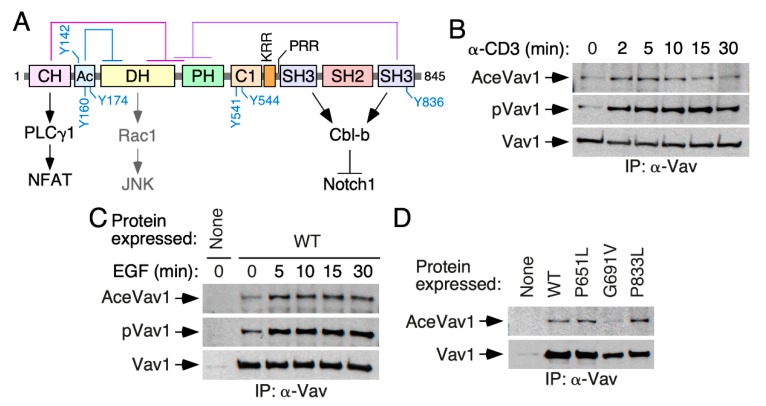
Vav1 lysine acetylation is stimulation- and SH2-dependent. (**A**) Schematic representation of the structure of Vav1. The intramolecular interactions that maintain the inactive, nonphosphorylated state of the protein are shown on top. The main regulatory phosphosites are shown in blue. The main downstream pathways are shown at the bottom. Abbreviations have been introduced in the main text. (**B**) Kinetics of Vav1 lysine acetylation (top panel) and tyrosine phosphorylation (middle panel) in T cell receptor (TCR)-stimulated Jurkat cells. The bottom panel shows the amount of endogenous Vav1 immunoprecipitated in each experimental time-point. Similar results were obtained in six independent experiments. Ace, acetylated; p, tyrosine-phosphorylated; IP, immunoprecipitation. (**C**) Kinetics of Vav1 lysine acetylation (top panel) and tyrosine phosphorylation (middle panel) in epidermal growth factor (EGF)-stimulated COS1 cells. The bottom panel shows the amount of ectopically expressed Vav1 immunoprecipitated in each experimental condition. Similar results were obtained in three independent experiments. (**D**) Levels of Lys acetylation (top panel) and Tyr phosphorylation (middle panel) of indicated Vav1 mutant proteins ectopically expressed in COS1 cells. The bottom panel shows the amount of immunoprecipitated Vav1 obtained in each case (*n* = 3).

Vav proteins contain a complex array of structural domains that include a calponin-homology (CH) domain, an acidic (Ac) region, the catalytic Dbl-homology (DH) domain, a pleckstrin-homology (PH) region, a C1-type zinc finger (C1) domain, a lysine-rich region (KRR), a proline-rich region (PRR), and a SH2 domain flanked by N- (NSH3) and C-terminal (CSH3) SH3 regions (Figure 1A). The noncatalytic domains play pleiotropic roles during signal transduction, contributing to the intramolecular regulation of Vav proteins (e.g., CH, Ac, PH, CSH3), the proper conformation of the central catalytic core (PH, C1), the phosphorylation step (SH2, CSH3), the stability at the plasma membrane (C1-KR motif), and the engagement of GTPase-independent routes (CH, SH3 domains) [4,5]. These functions are not mutually exclusive. For example, in the case of Vav1, its CH negatively controls the catalytic activity of the protein in cis and, at the same time, participates as an effector region that triggers the phospholipase Cγ- and Ca^2+^-dependent stimulation of NFAT [4,5]. Similarly, the Vav1 CSH3 participates in the optimal phosphorylation of the proteins downstream of the T cell receptor (TCR) as well as in the negative regulation of the Notch1 pathway [18,19].

The activity of Vav proteins is regulated by tyrosine phosphorylation-dependent conformational changes [4,5]. In the nonphosphorylated state, Vav proteins adopt a close conformation that is glued together by interactions of the Vav1 CH-Ac and the CSH3 regions with the central DH-PH cassette [20,21,22,23,24] (Figure 1A). These interactions occlude the effector surfaces of Vav proteins, leading to the inhibition of their catalytic and adaptor-like functions. Upon cell stimulation, the phosphorylation of Vav proteins on specific tyrosine residues (Figure 1A) leads to the release of those autoinhibitory interactions, the exposure of the effector sites of the molecule, and to full activation [20,21]. However, current evidence indicates that the biological activity of Vav proteins can be subjected to additional regulatory layers, including proteolytic cleavage, ubiquitinylation, and arginine methylation [4,5]. More recently, we have found that the signaling output of activated Vav1 proteins is modulated by the binding to membrane-resident phosphatidylinositol 5-phosphate. This binding, which favors the stability of Vav1 at the plasma membrane, is mediated by the coordinated action of the Vav1 C1 and KR regions [25]. The use of high-throughput mass spectrometry analyses has also revealed that Vav proteins become acetylated on a large number of lysine residues [26,27,28,29,30]. However, the functional impact of this posttranslational modification on the biological activity of these proteins is as yet unknown. Here, we show that lysine acetylation reconfigures the signaling diversification functions of Vav1 in T lymphocytes. Interestingly, this new regulatory layer is not conserved in nonhematopoietic cells and Vav1 paralogs.

## 2. Methods

### 2.1. Mammalian Expression Vectors

All the Vav family constructs used in this work encode versions of the murine species and were DNA sequence-verified in our Genomics Facility. Plasmids encoding wild-type Vav1 (Vav1^WT^) (pJLZ52), Vav1^Δ1−186^ (pMJC10), Vav1^Δ835−845^ (pSRF49), Vav1^G691V^ (pSRF46), Vav1^CAAX^ (pSRF93), Vav1^G691V+CAAX^ (pSRF96), Vav1^Y174E^ (pMB123), and EGFP-Vav1^WT^ (pSRM3) were previously described [21,24,25,31,32]. The pNFAT-Luc (Cat. number 17870) plasmid was obtained from Addgene (Watertown, MA, USA), the pSRE-luc (Cat. number 219081), the pFR-Luc and pFA2-cJun plasmids were obtained from Stratagene (now, Agilent Technologies, Santa Clara, CA, USA), and the pRL-SV40 plasmid was obtained from Promega (Madison, WI, USA). The rest of the plasmids encoding Vav1 mutant proteins were generated in this work by site-directed mutagenesis using the high-fidelity NZYProof DNA polymerase (Cat. number 14601; NZYTech, Lisbon, Portugal) and the appropriate combination of mutation-bearing oligonucleotides (Table S1).

### 2.2. Immunological Reagents

The rabbit polyclonal antibodies to the Vav1 DH domain (Lab reference number 302-5), phospho-Y^174^ (Lab reference number 613), phosphoY^280^ (Lab reference number 595), and phosphoY^836^ (Lab reference number 622) have been described elsewhere [21,32,33]. Other antibodies used in this study include those specific to human CD3 (UCHT1 clone, Cat. number 217570; Merk-Millipore, Burlington, MA, USA), Vav1 (Cat. number sc-8039; Santa Cruz Biotechnology, Dallas, TX, USA), phosphotyrosine (Cat. number sc-7020; Santa Cruz Biotechnology), acetyl-lysine (Cat. number AB3879; Merck-Chemicon, Temecula, CA, USA), CD98 (Cat. number ab108300; Abcam, Cambridge, UK), GAPDH (Cat. number sc-25778, Santa Cruz Biotechnology), polyhistidine (Cat. number H-1029; Sigma-Aldrich, Saint Louis, MO, USA), HA (Cat. number 5017; Cell Signaling Technology, Danvers, MA, USA) and tubulin α (Cat. number CP06; Merck-Calbiochem, Burlington, MA, USA). Rhodamine-labeled phalloidin (Cat. number PHDR1) was obtained from Cytoskeleton (Denver, CO, USA).

### 2.3. Cell Culture

Jurkat cells were obtained from the ATCC (Manassas, VA, USA) and grown in RPMI-1640 medium supplemented with 10% fetal calf serum, 1% L-glutamine, penicillin (10 μg/mL), and streptomycin (100 μg/mL). COS1 cells were obtained from the ATCC and grown in DMEM supplemented with 10% fetal calf serum, 1% L-glutamine, penicillin (10 μg/mL), and streptomycin (100 μg/mL). All tissue culture reagents were obtained from Gibco-Thermo Fisher Scientific (Waltham, MA, USA). All cell lines were maintained at 37 °C in a humidified, 5% CO_2_ atmosphere. When required, cells were stimulated for the indicated periods of time with antibodies to human CD3 (7.5 μg/mL, Cat. number 217570, Merck; Jurkat cells) and epidermal growth factor (EGF) (50 ng/mL, Cat. number E9644, Sigma-Aldrich; COS1 cells). In the latter case, cells were previously starved without serum for 3 h.

### 2.4. Immunoprecipitations

In the case of COS1 cells, we ectopically expressed the indicated proteins using the diethylaminoethyl-dextran (10 mg/mL, Cat. number D9885, Sigma-Aldrich)/chloroquine (2.5 mM, Cat. number C6628, Sigma-Aldrich) method [34]. Two 10-cm diameter plates were transfected per condition using 10 μg of plasmid per plate. At 48 h post-transfection, cells were washed in a phosphate-buffered saline solution and lysed with the aid of a scrapper in 1 mL of RIPA buffer [10 mM Tris-HCl (pH 7.5), 150 mM NaCl, 1% Triton X-100 (Cat. number X100, Sigma-Aldrich), 1 mM Na_3_VO_4_ (Cat. number S6508, Sigma-Aldrich), 1 mM NaF (Cat. number S7920, Sigma-Aldrich), and the CØmplete protease inhibitor cocktail (Cat. number 11836145001; Roche, Basel, Switzerland). Cellular extracts were kept 5 min on ice and subsequently centrifuged at 14,000 rpm for 10 min at 4 °C to eliminate cell debris. Then, the supernatants were incubated with either 7 μL of an antibody to Vav1 or 5 μL of an antibody to acetylated lysine residues overnight at 4 °C. Then, immunocomplexes were collected with Gammabind G-Sepharose beads (Cat. number GE17-0885-01; GE Healthcare, Chicago, IL, USA), washed three times in RIPA buffer, resuspended in SDS-PAGE buffer, boiled for 5 min, electrophoresed, and subjected to immunoblot analyses with the indicated antibodies.

In the case of Jurkat, 10 × 10^6^ cells were transfected with 20 μg of DNA per condition using the Program #5 of the Neon transfection system (Cat. number MPK5000; Invitrogen, Carlsbad, CA, USA) according to the manufacturer’s instructions. After 48 h, the cells were washed, transferred to a 1.5 mL Eppendorf tube and, after low-speed centrifugation at 4 °C, resuspended in lysis buffer with extensive vortexing. The cell extracts obtained were processed as above. However, in the case of immunoprecipitation experiments using GFP-Vav1, the cell lysates were incubated with the GFP-Trap reagent (Cat. number gta-100; ChromoTek, Planegg, Germany) for 1 h at 4 °C. Washed immunocomplexes were collected by centrifugation and subjected to immunoblot analyses as above. In all cases, total cellular lysates were analyzed in parallel to monitor the expression of the ectopically expressed proteins used in each experiment.

### 2.5. Western Blotting and Quantitation

Immunoblot analyses were carried out as described elsewhere [32]. When appropriate, the acetylation and phosphorylation levels of Vav1 in specific experimental conditions were calculated by densitometry analyses using ImageJ (NIH; Bethesda, Rockville, MD, USA). In all cases, the densitometry values for the specific posttranslational modification of Vav1 were normalized considering the total amount of immunoprecipitated protein obtained in each condition. The acetylation or phosphorylation levels found for Vav1^WT^ in nonstimulated cells were given an arbitrary value of 1 in each case.

### 2.6. Mass Spectrometry Analyses

Upon the GFP-trap-mediated immunoprecipitation, the GFP-Vav1 proteins were separated by one-dimensional SDS-PAGE electrophoresis and Coomassie blue-stained. The area of the gel containing the GFP-Vav1 (according to molecular weight mobility) was excised and subjected to in-gel digestion with trypsin following a modified version of a protocol described elsewhere [35]. To this end, the gel pieces were destained at 37 °C for 15 min using a solution of 50% acetonitrile in 50 mM sodium bicarbonate. Subsequently, the protein reduction and alkylation steps were performed using 10 mM DTT (56 °C, 45 min) and 55 mM iodoacetamide (room temperature, 30 min). Upon digestion with trypsin (6.25 ng/mL) at 37 °C for 18 h, the peptide-containing solutions were acidified with formic acid (FA) and desalted by using C18-Stage-Tips columns [36], partially dried and stored at –20 °C. For mass spectrometry analysis, the peptides were dissolved in 0.5% FA/3% acetonitrile (ACN), loaded onto a trapping column (nanoACQUITY UPLC 2G-V/M Trap Symmetry 5 μm particle size, 180 μm × 20 mm C18 column; Cat. number 186006527; Waters Corp., Milford, MA, USA), and separated using a nanoACQUITY UPLC BEH C18 column (1.7 μm, 130 Å and a 75 μm × 250 mm; Cat. number 186003545, Waters Corp.) at 40 °C and with a linear gradient ranging from 7% to 35% of solvent B (ACN/0.1% FA; flow rate: 300 nL/min over 30 min and 5 min to 55%). The LTQ-Orbitrap Velos (Thermo Fisher Scientific) was operated in the positive ion mode applying a data-dependent automatic switch between survey mass spectra (MS) scan and tandem mass spectra (MS/MS) acquisition. MS scans were acquired in the mass range of m/z 400 to 1400 with 30,000 resolution at m/z 400, with a lock mass option enabled for the 445.120025 ion [37]. The 10 most intense peaks having ≥2 charge state and above 500 intensity threshold were selected for fragmentation by high-energy collision-induced dissociation (HCD) at 42% normalized energy, 100 ms activation time, and 2 m/z precursor isolation width. The maximum injection time was 1000 ms and 500 ms for survey and MS/MS scans, respectively. Automatic gain control was 1 × 106 for MS and 5 × 10^4^ for MS/MS scans. Dynamic exclusion was enabled for 90 s. MS/MS spectra were acquired in the Orbitrap with 7500 resolution. A parent mass list with m/z corresponding to possible acetylated peptides was built using Skyline and included in the acquisition method [38]. In order to avoid expanding the list above the equipment limit of 2000 entries, only species with a maximum of two missed cleavages and three charges were included in the fragmentation list.

Mass spectra were analyzed using the SEQUEST HT algorithm of the Proteome Discoverer software (Cat. number OPTON-30795, Thermo Fisher Scientific). All tandem mass spectra were searched against a custom database containing Uniprot complete mouse sequences and Mann contaminants. The search parameters were fully tryptic digestion with a maximum of two missed cleavages, 20 ppm, and 0.02 Da mass tolerances for precursor and product ions respectively, carbamidomethylated cysteines, variable oxidation of methionine, and variable acetylation on lysine (+ 42.011 Da). A 1% false discovery rate using Percolator [36] was used for peptide validation. In order to evaluate the proportion of acetylated peptides, a mass deviation of 2 ppm was used for precursor ion area detection, and peptides with a 5% false discovery rate were also included in the calculation. Spectra of peptides identified as acetylated were manually inspected for the presence of acetylation marker ions [38,39].

The abundance of the peptides was determined by integration of the total ion current of the MS signal of each peptide m/z. For the estimation of the proportion of acetylated peptides, the abundance of all the peptide species containing the same modified lysine residue was added and compared with the total abundance of the peptide species containing the unmodified lysine residue.

### 2.7. Phylogenetic Conservation Analyses

Amino acid sequences from the Vav family proteins were obtained from the UniProt database (National Center for Biotechnology Information, Bethesda, MD, USA) and aligned using the multiple sequence alignment by log-expectation algorithm in the Jalview software [40,41,42].

### 2.8. 3D Structures

Three-dimensional (3D) structures were generated in PyMol and the Protein Data Bank-stored crystal structure files for the Vav1-Rac1 complex (3BJI), the Vav1 CH-Ac-DH-PH-C1 (3KY9), the Vav1 KRR-NSH3 (1K1Z), the Vav1 SH2 (2MC1), and the Vav1 CSH3 (2KBT) regions.

### 2.9. Luciferase Reporter Assays

Exponentially growing Jurkat cells (2 × 10^7^ cells per condition) were electroporated (250 V, 950 μF pulses) using a Gene Pulser II apparatus (Cat. number 165-2106; BioRad, Hercules, CA, USA). In the case of c-Jun N-terminal kinase (JNK) assays, the cells were electroporated with the firefly luciferase reporter pFR-Luc (5 μg), pFA2-c-Jun (2 μg), a vector constitutively expressing the Renilla luciferase (pRL-SV40, 10 ng), and 20 μg of the indicated expression vector. In the case of nuclear factor of activated T cells (NFAT) assays, the cells were electroporated with 3 μg of a luciferase reporter plasmid containing three NFAT binding sites (pNFAT/luc), 10 ng of pRL-SV40, and the indicated experimental plasmids as above. When required, empty plasmids were included in the electroporations to maintain constant the total amount of DNA introduced in cells among all the experimental samples. After 36 h in complete medium, the electroporated cells were either left untreated or stimulated with the indicated antibodies for seven hours. Then, cells were washed in serum-free media, lysed in Passive Lysis Buffer (Cat. number E1960; Promega), and the luciferase activity obtained in each condition was recorded using the Dual-Luciferase Reporter System (Cat. number E1960, Promega) according to the supplier’s recommendations in a Lumat LB 9507 luminometer (Berthold Technologies, Bad Wildbad, Germany). The raw values obtained were normalized according to the activity of the *Renilla* luciferase recorded in each sample. Final values are represented as the fold change of the normalized luciferase activity obtained when compared to the control sample. In all cases, the abundance of the ectopically expressed proteins under each experimental condition was verified by analyzing the aliquots of the cell extracts used in the luciferase experiments by immunoblot.

### 2.10. Rac1 GTPase Activation Assays

Total cellular lysates were obtained as above, snap frozen, thawed, quantified for total protein concentration, and analyzed using Rac1 G-LISA^®^ assay kits according to the manufacturer’s instructions (Cat. number BK135, Cytoskeleton).

### 2.11. Statistical Analyses

All the statistical analyses were carried out using GraphPad Prism software (version 6.0 and 8.0; San Diego, CA, USA). The number of replicates and the statistical test used in each case are indicated in the figure legends. In all cases, the *P* values have been depicted using the * (when < 0.05), ** (when < 0.01), and *** (when < 0.001) notation.

## 3. Results

### 3.1. Acetylation of Vav1 is Modulated by Upstream Stimuli

Given the available proteomics data [26,27,28,29,30], we investigated whether the acetylation of Vav1 on lysine residues takes place in either a constitutive or a stimulus-dependent manner. To this end, we first used antibodies to acetylated lysine residues to follow the evolution of the acetylation state of the endogenous Vav1 protein in TCR-stimulated Jurkat cells. As shown in Figure 1B (top panel) and Table S2, Vav1 shows some basal acetylation in naïve cells. However, upon TCR stimulation, the protein undergoes a rapid and transient increase in the lysine acetylation levels. These kinetics mimic those found in the case of the phosphorylation of Vav1 on tyrosine residues under the same experimental conditions (Figure 1B, second panel from top; Table S2). The reblotting of the same blots with antibodies to Vav1 confirmed similar amounts of the immunoprecipitated protein in each of the time points interrogated in these experiments (Figure 1B, bottom panel). Similar results were observed when the acetylation of ectopically expressed Vav1 was monitored upon the stimulation of serum-starved COS1 cells with epidermal growth factor (EGF) (Figure 1C and Table S2). The acetylation of Vav1 is not impaired by inactivating mutations in any of its two SH3 domains (Figure 1D and Table S2). By contrast, it is diminished when the Vav1 SH2 is inactivated by a point mutation (G691V) (Figure 1D and Table S2). These results indicate that, similarly to the tyrosine phosphorylation step, the lysine acetylation of Vav1 is both stimulation- and SH2-dependent.

### 3.2. Vav1 is Acetylated on Multiple Lysine Residues

**Figure 2:**
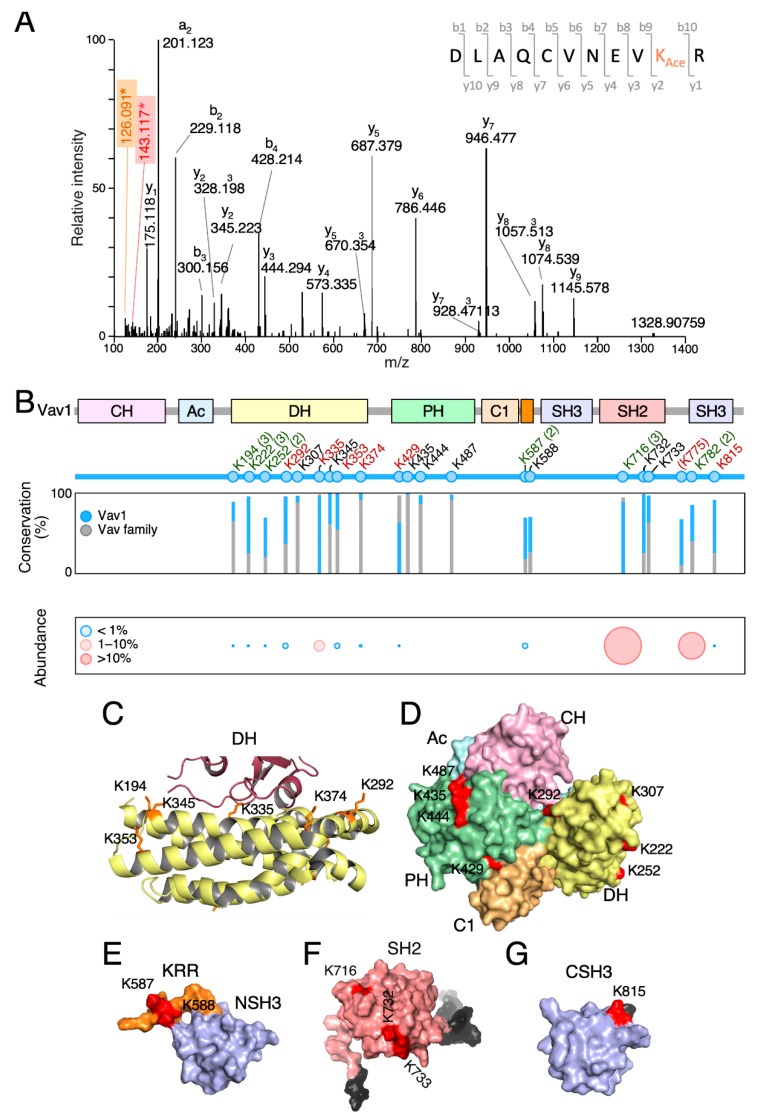
Vav1 is acetylated on multiple lysine residues. (**A**) Example of the tandem mass spectrometry spectrum of a peptide DLAQCVNEVK^374^_Ace_R containing the acetylated (Ace) Lys^374^ residue. The plot shows the acetylated lysine immonium ion at mass-to-charge (m/z) ratio of 143 (red box) and its derivative displaying an m/z ratio of 126 (brown box, generated by loss of ammonia). (**B**) Top, main Vav1 lysine acetylation sites identified by mass spectrometry. The new acetylation sites found in our study are shown in red. Residues found in our study and previous reports are indicated in green. The number of times in which these acetylation sites has been detected before this study is shown in brackets. If only once, the number is not indicated. Previously identified residues that were not found in our analyses are indicated in black. Lys^775^ (shown between round brackets) is not conserved in human VAV1. Middle, phylogenetic conservation of the indicated acetylated residues in all Vav1 analogs (blue bars) and paralogs (gray bars) identified to date. Bottom, levels of acetylation of indicated residues found in vivo. (**C**) Three-dimensional (3D) structure of the Vav1 Dbl-homology (DH) domain (yellow) and part of the Rac1 switch regions (brown). The side chains of the indicated lysine residues are shown in orange color. (**D**) 3D structure of the calponin-homology (CH)-acidic (Ac)-DH-pleckstrin-homology (PH)-C1-type zinc finger (C1) regions of Vav1 in the inactive conformation. Acetylated residues are shown in red. (**E**–**G**) Three-dimensional (3D) structure of the Vav1 lysine-rich region (KRR) (orange) and C-terminal (CSH3) domain (**E**), the Vav1 SH2 (**F**), and the Vav1 CSH3 domain of Vav1 (**G**) with indicated acetylated residues shown in red. In F and G, N- and C-terminal extensions of the domains are indicated in gray. In G, the Lys^782^ residue could not be depicted since it is not included in the available 3D structure.

The key regulatory phosphosites of Vav proteins become phosphorylated upon the stimulation of multiple upstream receptors and cell types [4,5]. To investigate the level of conservation of the Vav1 lysine acetylation landscape across cell types, we next carried out mass spectrometry analyses using immunoprecipitated Vav1 obtained from exponentially growing, nonstimulated COS1 cells. Tryptic peptides were considered to harbor acetylated lysine residues when they fulfilled two concurrent criteria: **(i)** the presence of diagnostic ions with a 126.1 to 143.1 mass-to-charge ratio [43], and **(ii)** a delay in the retention time when compared to the nonacetylated counterpart. An example of such an identification is shown in Figure 2A for the acetylated version of the Lys^374^ residue.

Using this approach, we identified six acetylated residues that were already found in previous high-throughput proteomic experiments (Lys^194^, Lys^222^, Lys^252^, Lys^587^, Lys^716^, and Lys^782^) (Figure 2B, residues in green) [26,27,28,29]. In addition, we found seven new residues that had not been detected before (Lys^292^, Lys^335^, Lys^353^, Lys^374^, Lys^429^, Lys^775^, and Lys^815^) (Figure 2B, residues in red color). However, eight acetylation sites described in previous studies were not found in our analyses (Lys^307^, Lys^345^, Lys^435^, Lys^444^, Lys^487^, Lys^588^, Lys^732^, and Lys^733^) (Figure 2B, black residues) [26,27,28,29,30]. These missing residues have not been recurrently found in previous studies either [26,27,28,29,30], suggesting that they could represent either very poorly acetylated residues or sites whose detection is highly dependent on the analytical techniques used. In our case, we believe that the latter reason could apply for the following reasons: (i) to avoid using a m/z list above the equipment limit (2000 entries), we only included in the fragmentation list m/z species with a maximum of two missed cleavages and three charges. Using this threshold, previously described peptides containing the acetylated lysine residues located at positions 345, 444, 487, 732, and 733 [27,30] could have been missed in our analyses; (ii) other acetylated residues (e.g., Lys^307^) [27] could have been overlooked due to oxidation of the acetylated peptide.

All the identified acetylation sites are surfaced-exposed in the Vav1 3D structure (Figure 2C–G). They also cluster within relevant domains of the protein such as the DH Rac1 binding site (Figure 2B,C), the DH surface opposite to the catalytic site (Figure 2B–D), the PH (Figure 2B,D), the KRR (Figure 2B,E), the SH2 (Figure 2B,F), and the CSH3 (Figure 2B,G). However, they display in general low levels of phylogenetic conservation among the Vav1 analogs and paralogs identified to date in both invertebrate and vertebrate species (Figure 2B and Figure S1) [5].

According to the levels of in vivo acetylation determined in the foregoing mass spectrometry analyses, we found residues that were poorly (less than 1% of the total protein; e.g., Lys^194^, Lys^222^, Lys^252^, Lys^292^, Lys^353^, and Lys^587^), mildly (10% of the total protein; e.g., Lys^335^), and highly (20%–35% of the total protein; e.g., Lys^716^, Lys^782^) acetylated under basal conditions in vivo (Figure 2B, bottom panel).

### 3.3. Rationale Used for the Characterization of Acetylation Sites

Given the large number of residues involved, we decided to characterize a selection of the Vav1 acetylation sites that fulfilled a number of criteria: (i) detection in more than one mass spectrometry study (Lys^222^, Lys^252^, Lys^587^, Lys^716^, and Lys^782^; Figure 2B); (ii) for those detected in a single study, localization on a potentially important region of the protein (Lys^335^ and Lys^374^; Figure 2B) or in the neighborhood of one of sites that were found in previous proteomics experiments (Lys^588^; Figure 2B,E); and (iii) level of conservation when compared with Vav1 homologs (Figure 2B and Figure S1). To investigate the relevance of those acetylation sites, we mutated each of them into either Arg or Gln. These mutations are usually utilized in protein acetylation studies to mimic the constitutive deacetylated and acetylated states of lysine residues, respectively [26,44,45].

**Figure 3:**
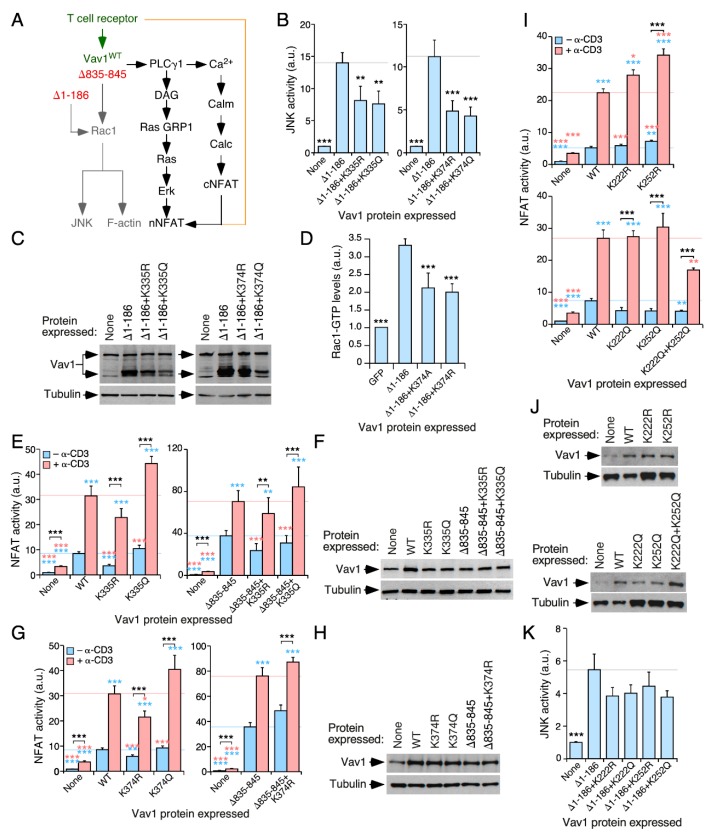
The acetylation of DH domain-localized lysine residues modifies Vav1 signaling outputs. (**A**) Schematic representation of the Vav1-dependent pathways that have been characterized in T cells. The Rac1-dependent pathway directly stimulated by the catalytic activity of Vav1 is shown in gray. The Vav1 adaptor-like pathway involved in the activation of nuclear factor of activated T cells (NFAT) is shown in black. Synergies established with parallel, TCR/CD3-triggered pathways are shown in brown. The wild-type (WT) and constitutively active versions of Vav1 used in this study are shown in green and red, respectively. DAG, diacylglycerol; GRP, GDP-releasing factor; Calm, calmodulin; Calc, calcineurin; cNFAT, cytosolic NFAT; nNFAT, nuclear NFAT. (**B**) Levels of stimulation of c-Jun N-terminal kinase (JNK) triggered by the indicated Vav1 proteins in nonstimulated Jurkat cells. Data represent the mean ± SEM. **, *p* < 0.01; ***, *p* < 0.001 using the Mann–Whitney U test of indicated experimental values compared to Vav1^Δ1−186^-expressing cells (n = three independent experiments, each performed in triplicate). (**C**) Representative immunoblots showing the abundance of the ectopic Vav1 proteins and endogenous tubulin α in the experiments shown in B. (**D**) The activation of endogenous Rac1 upon the expression of indicated Vav1 proteins in COS1 cells. (**E**,**G**,**I**) Activation levels of NFAT elicited by the indicated Vav1 proteins in nonstimulated and TCR-stimulated Jurkat cells. Data represent the mean ± SEM. Statistical values were obtained using the Mann–Whitney U test. Blue and salmon asterisks indicate the significance level compared with nonstimulated and TCR-stimulated Vav1^WT^-expressing cells (left panel) or Vav1^Δ835−845^-expressing cells (right panel), respectively (n = 3 independent experiments, each performed in triplicate). *, *p* < 0.05; **, *p* < 0.01; ***, *p* < 0.001. (**F**,**H**,**J**) Representative immunoblots showing the abundance of the ectopic Vav1 proteins and endogenous tubulin α in the experiments shown in E (**F**), G (**H**), and I (**J**). Endogenous tubulin α was used as loading control in all cases (**F**,**G**,**I**; bottom panels). (**K**) Activation of JNK by the indicated ectopically-expressed Vav1 proteins (bottom) in nonstimulated Jurkat cells. Data represent the mean ± SEM. ***, *p* < 0.001 using the Mann–Whitney U test. *n* = 3 independent experiments, each performed in triplicate.

Then, we tested the biological activity of each of those mutant proteins using luciferase reporter-based c-Jun N-terminal kinase (JNK) and NFAT activity assays in Jurkat cells. Vav1 activates JNK in a catalysis- and Rac1-dependent manner [21,24,32]. Therefore, the activity of this downstream kinase is traditionally used as a surrogate biological readout to measure the activation levels of the Vav1 catalysis-dependent pathways (Figure 3A) [21,32]. Depending on the experiment, we used in these experiments either the wild-type (Vav1^WT^) or a constitutively-active (Vav1^Δ1−186^) version of Vav1. Vav1^Δ1−186^ exhibits tyrosine-phosphorylation activity owing to the lack of the autoinhibitory N-terminal CH and Ac regions (Figure 3A) [24]. As a result, it yields higher levels of activation of the Rac1-JNK pathway than the wild-type counterpart. However, due to the lack of the CH region, this protein cannot activate the NFAT pathway [21,24] (Figure 3A).

The stimulation of the NFAT pathway by Vav1 is mediated by a GTPase-independent, adaptor-like mechanism that involves the phospholipase Cγ-mediated stimulation of the nuclear translocation and activation of this transcriptional factor (Figure 3A). The optimal activation of this pathway requires synergistic inputs from TCR-triggered signals (Figure 3A). Due to this, the stimulation of NFAT is boosted by the engagement of the TCR even when using constitutively active versions of Vav1. Due to this, this assay is conventionally used to monitor the activation levels of this adaptor function of Vav1 [46,47,48]. In this assay, the mutations in the acetylation sites were generated both in the case of the full-length protein and one of its C-terminally truncated versions (Vav1^Δ835−845^). The latter protein displays, similarly to Vav1^Δ1−186^, constitutive, tyrosine phosphorylation-independent biological activity due to the lack of the autoinhibitory CSH3 domain [21]. However, unlike the case of Vav1^Δ1−186^ [21,24,32], Vav1^Δ835−845^ is capable of activating the NFAT pathway since it still keeps the N-terminal CH domain [21]. The use of this mutant allowed us to discriminate whether the alterations generated by the acetylation site mutations on Vav1 signaling were related to either the early phosphorylation-dependent Vav1 activation step (in that case, the mutants should affect the biological activity of Vav1^WT^ but not of Vav1^Δ835−845^) or to subsequent effects in the effector signaling phases of the activated protein (in this case, the mutations on the acetylation sites must also affect the signaling output of Vav1^Δ835−845^).

We considered that a lysine acetylation site was functionally relevant when it fulfilled two criteria: **(i)** that the Lys to Arg or the Lys to Gln mutant versions of Vav1 on those sites elicited changes in either the NFAT or JNK activity when compared to the WT counterpart; **(ii)** that those effects were opposite in the case of the Lys to Arg and Lys to Gln mutants (e.g., effect/no effect, activation/inactivation, or vice versa); and (iii) although prima facie counterintuitive, we also deemed as potentially relevant sites those in which the Lys to Arg and Lys to Gln mutations elicited the same effect on the biological activity of the mutant protein. The inclusion of these sites was based on the fact that those two types of mutations can elicit the same effect if they target lysyl groups that perform a key intramolecular role in the protein that cannot be replaced by the side group of the newly incorporated mutant residue. This scenario was contemplated given our previous experience with the characterization of the key regulatory tyrosine phosphorylation sites of Vav proteins. Indeed, during these analyses, we found that the dephosphorylation- (Tyr to Phe) and phosphorylation-mimicking (Tyr to Glu) mutations elicited in fact the same activation effect on the mutant proteins [20,21,24,33] (see our further explanation of this in the Discussion section).

### 3.4. The Acetylation of Lysine Residues Located in the DH Domain Contribute to the Regulation of Vav1 Activity

Using the foregoing criteria, we first focused on Lys^335^ and Lys^374^. These residues are located within the catalytic site of the DH domain (Figure 2B,C). Lys^374^ was particularly interesting, since it is known that it can establish hydrogen bonds with residues located in the switch II region of the bound GTPase [49,50]. When tested in JNK assays, we found that the targeting of any of those two lysine residues leads to a marked reduction in the biological activity of the constitutively active Vav1^Δ1−186^ (Figure 3B,C). This inhibitory effect is seen in versions of Vav1^Δ1−186^ bearing either Lys to Arg or Lys to Gln mutant versions of these residues (Figure 3B,C). However, we also found the same effect when the Lys^374^ residue was mutated into other amino acids such as Ala (data not shown). Similar data were observed when these mutants were tested in other catalysis-dependent readouts such as the stimulation of serum-responsive factor and the AP1 family (data not shown). This is probably connected with the reduced catalytic output of these mutant proteins, as assessed by the determination of Vav1-driven levels of GTP-bound Rac1 in cells using G-LISA^®^ methods (Figure 3D).

When tested in NFAT assays, we found that the Vav1^K335R^ (Figure 3E, left panel; Figure 3F) and the Vav1^K335Q^ (Figure 3E, left panel; Figure 3F) mutants promote a slight reduction and elevation in the stimulation of this transcriptional factor when compared to Vav1^WT^, respectively. The effect of those mutations is lost when tested in the context of the constitutively active Vav1^Δ835−845^ protein (Figure 3E, right panel; Figure 3F). Similar results were obtained when using the two mutant versions of the Lys^374^ residue (Figure 3G,H). Given that the Lys^335^ and Lys^374^ residues are not located within the effector CH domain, these results suggest that the effect derived from the mutation of those sites has to be related to conformational changes in the full-length protein that affect the exposure of the effector interface of the CH domain. A similar effect has been observed before when analyzing mutations targeting key Ac-, C1-, and CSH3-located phosphosites [21,32]. These results indicate that the acetylation of these sites can modulate the catalytic (reduction) and adaptor (elevation) outputs of Vav1 (see the Discussion section).

Next, we tested the functional role of the acetylation sites (Lys^222^, Lys^252^) present in a region of the DH domain that is located opposite to the catalytic site (Figure 2B–D). We observed that the deacetylation-mimicking K222R and K252R mutants promote a very slight, although statistically significant increase in the activity of the full-length Vav1 protein to stimulate the NFAT pathway both in nonstimulated and TCR-stimulated cells (Figure 3I,J; upper panels). By contrast, the acetylation-mimicking K222Q and K252Q mutants did not elicit any statistically significant change in the activity of the full-length Vav1 toward NFAT (Figure 3I,J; lower panels). However, when combined together, the double K222Q+K252Q mutant causes a significant reduction in the activity of full-length Vav1 both in nonstimulated and stimulated cells in the NFAT assays (Figure 3I,J; lower panels). However, this compound mutation does not elicit any change in the activation levels of the JNK by Vav1^Δ1−186^ (Figure 3K). Thus, these two residues do seem to contribute cooperatively to the downregulation of the adaptor function of Vav1 that leads to the stimulation of the transcriptional factor NFAT.

### 3.5. The Acetylation of the KRR-Localized Lys^587^ Residue Affects Vav1 Signaling Output

**Figure 4:**
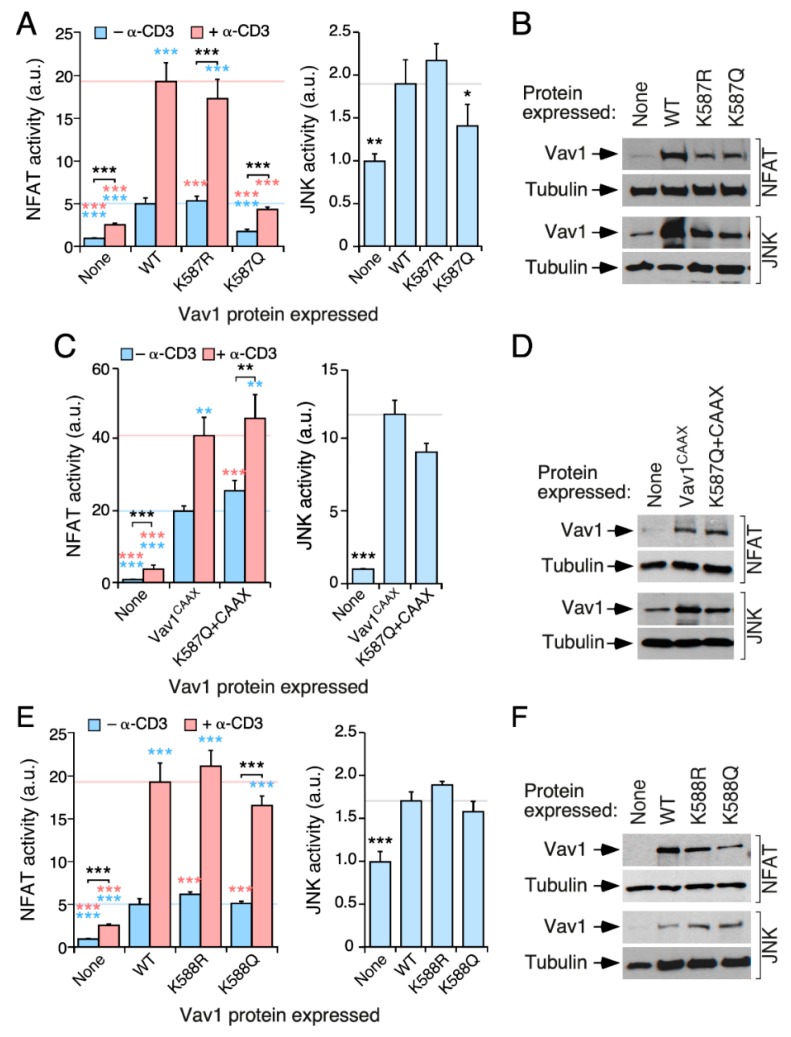
The acetylation of the Lys^587^ residue blocks the activation of NFAT by Vav1. (**A**,**C**,**E**) Activation of NFAT (**A**,**C**,**E**; left panels) and JNK (**A**,**C**,**E**; right panels) by indicated Vav1 proteins in nonstimulated and TCR-stimulated Jurkat cells. Data and *p* values are depicted as in Figure 3E (*n* = 3 independent experiments, each performed in triplicate). (**B**,**D**,**F**) Representative immunoblots showing the abundance of the ectopic Vav1 proteins in the experiments shown in A (**B**), C (**D**), and E (**F**). Endogenous tubulin α was used as loading control in all cases. *, *p* < 0.05; **, *p* < 0.01; ***, *p* < 0.001.

We next analyzed the potential contribution of the acetylation of the Lys^587^ and Lys^588^ residues to the biological activity of Vav1. These two residues are potentially interesting, since they are located in the Vav1 KRR that mediates interactions with phosphatidylinositol monophosphates [25] (Figure 2B,E). We found that the K587Q mutation (that mimics the constitutively acetylated state of this residue), but not the K587R missense change (that mimics the deacetylated state of this site), promotes a marked downregulation of the NFAT activity of the full-length Vav1 protein when tested both in nonstimulated and TCR-stimulated Jurkat cells (Figure 4A, left panel; Figure 4B, two top panels). It also induces a slighter inhibition of full-length Vav1-triggered JNK activity (Figure 4A, right panel, Figure 4B, two bottom panels). The negative effect of the K587Q mutation is eliminated when tested in the context of the Vav1^CAAX^ protein (Figure 4C,D), thus indicating that the defect is related with the stable association of the mutant protein with the plasma membrane. The same behavior was observed before in the case of mutants targeting other residues of the Vav1 KRR [25]. The activity of Vav1^K588R^ and Vav1^K588Q^ proteins show no statistically significant alteration when tested in both NFAT and JNK assays (Figure 4E,F). This result indicates that the Lys^587^ residue is a functionally relevant Vav1 acetylation site.

### 3.6. The Acetylation of the SH2-Located Lys^716^ Residue Limits Vav1 Signaling Output

**Figure 5:**
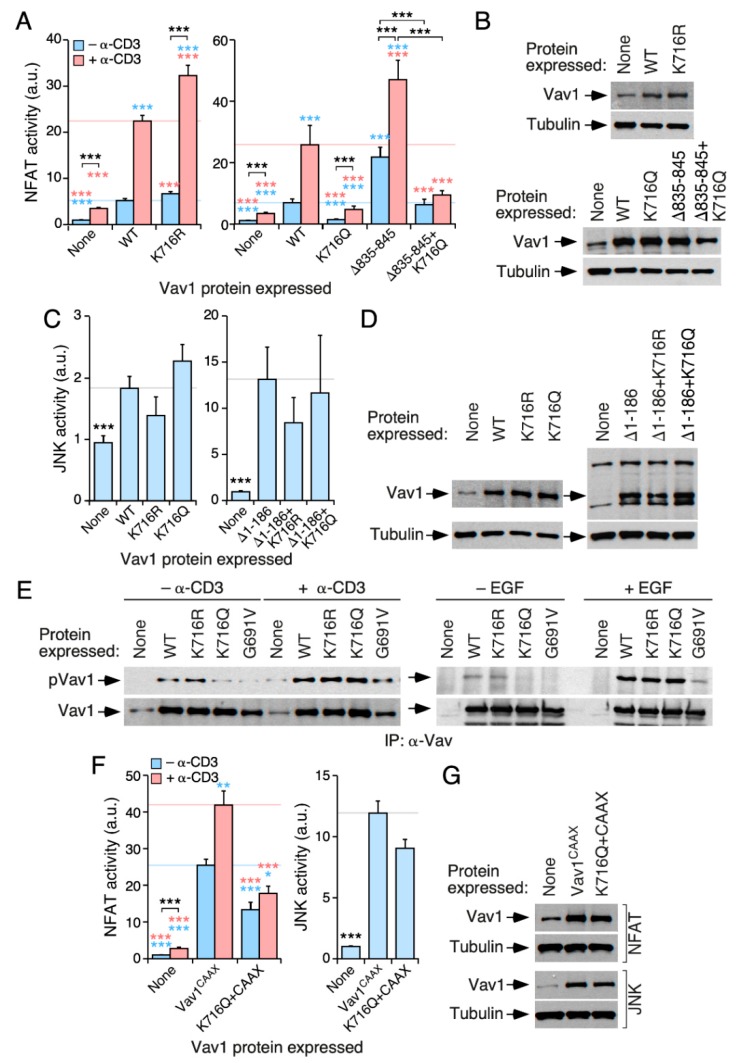
The acetylation of Lys^716^ impairs the activation of NFAT by Vav1. (**A**,**C**) Activation of NFAT (**A**) and JNK (**C**) by the indicated Vav1 proteins in nonstimulated and TCR-stimulated Jurkat cells. Data and *P* values are depicted as in Figure 3E (*n* = 3 independent experiments, each performed in triplicate). (**B**,**D**) Representative immunoblots showing the abundance of the indicated Vav1 proteins in the experiments shown in A (**B**) and C (**D**). Endogenous tubulin α was used as loading control in all cases. **(E)** Levels of tyrosine phosphorylation of the indicated Vav1 proteins immunoprecipitated from nonstimulated and stimulated Jurkat (top panels on the left) and COS1 (top panels on the right) cells. The stimulation conditions are indicated on top. The bottom panels show the amount of endogenous Vav1 immunoprecipitated in each sample. Similar results were obtained in three independent experiments. (**F**) Activation of NFAT (left panel) and JNK (right panel) by indicated Vav1 proteins in Jurkat cells under nonstimulation and TCR-mediated stimulation conditions. Data and *p* values are depicted as in Figure 3E (*n* = 3 independent experiments, each performed in triplicate). (**G**) Representative immunoblots showing the abundance of the ectopic Vav1 proteins in the transfected cells used in F. Endogenous tubulin α has been used as loading control. *, *p* < 0.05; **, *p* < 0.01; ***, *p* < 0.001.

The Lys^716^ residue is prima facie appealing because of the following: (i) it shows high levels of acetylation in cells according to our mass spectrometry determinations (Figure 2B); (ii) it is the only acetylation site conserved in all mammalian Vav family proteins (Figure 2B and Figure S1); and (iii) it is located in the vicinity of the grove of the Vav1 SH2 domain that participates in the interaction with tyrosine-phosphorylated motifs (Figure 2F). The mutations of this residue promote an increase (in the case of the deacetylation-mimicking Lys to Arg mutation) and a reduction (in the case of the acetylation-mimicking Lys to Gln mutation) on Vav1-triggered NFAT activity in both nonstimulated and TCR-stimulated cells (Figure 5A,B). The same effect is observed when the K716Q mutations are analyzed in the context of the hyperactive, phosphorylation-independent Vav1^Δ835−845^ version (Figure 5A, right panel; Figure 5B, two bottom panels). By contrast, the K716R and the K716Q versions of full-length Vav1 (Figure 5C,D; left panels) and Vav1^Δ1−186^ (Figure 5C,D; right panels) exhibit normal levels of JNK activation when compared to the appropriate controls. Thus, similarly to the Lys^222^, Lys^252^, and Lys^587^ sites (Figure 3 and Figure 4), the acetylation of the Lys^716^ residue specifically impairs the Vav1-mediated activation of the NFAT pathway.

Given the importance of the SH2 domain for the tyrosine phosphorylation of Vav1 [4,5], we next investigated the possible impact of the Lys^716^ mutations on Vav1 tyrosine phosphorylation levels. We found that the K716Q mutation, but not the K716R one, promotes a marked reduction in the tyrosine phosphorylation levels of full-length Vav1 in both nonstimulated Jurkat (Figure 5E, left panel) and serum-starved COS1 (Figure 5E, right panel) cells. This effect is similar to that found when using a Vav1 mutant protein bearing a point mutation (G691V) that inactivates the phosphotyrosine-binding activity of its SH2 domain (Figure 5E). However, unlike the case of the G691V missense change, the full-length Vav1 protein bearing the K716Q mutation exhibits levels of tyrosine phosphorylation comparable to those found in Vav1^WT^ when monitored in both TCR-stimulated Jurkat (Figure 5E, left panel) and EGF-stimulated COS1 (Figure 5E, right panel) cells. These results suggest that this mutation reduces, but does not abrogate, the affinity of the Vav1 SH2 domain for its tyrosine phosphorylated ligands. However, it is unlikely that this is the underlying cause of the deleterious effects of the K716Q mutation on Vav1 activity for the following reasons. (i) The K716Q mutation does not impair the stimulation of JNK by the full-length Vav1 protein (Figure 5C,D), which is an activity that is also tyrosine phosphorylation-dependent [21,33]. (ii) This mutation also impairs the stimulation of NFAT by the phosphorylation-independent Vav1^Δ835−845^ protein (Figure 5A,B). (iii) The reduced activity of the Vav1^K716Q^ mutant in NFAT assays cannot be restored by the attachment to the protein of the membrane-anchoring signal of H-Ras (Figure 5F,G). This signal promotes high levels of Vav1 activity due to enhanced membrane localization and phosphorylation [24].

Given that this acetylation site is conserved in both Vav2 (Lys^718^ residue) and Vav3 (Lys^717^ residue) (Figure S1), we next investigated whether the acetylation of this residue could influence the activity of other family proteins. We focused our attention on Vav3, given that Vav2 cannot stimulate the NFAT pathway in Jurkat cells [51]. We could only find minor effects of the K717Q mutation on the biological activity of full-length Vav3 in both nonstimulated and TCR-stimulated cells (Figure S2A,B). Moreover, the K717R mutation does not alter the signaling output of Vav3 (Figure S2A,B). Interestingly, we found that the impact of each of those mutations on the overall tyrosine phosphorylation levels of Vav3 and Vav1 are quite different. Thus, unlike the case of Vav1 (Figure 5E), we could not find any significant variation in the levels of phosphorylation between Vav3^WT^ and the Vav3^K717Q^ protein. Furthermore, the Vav3^K717R^ mutant unexpectedly exhibits much higher levels of phosphorylation than Vav3^WT^ (Figure S2C). Thus, despite its conservation (Figure S1), the Lys^716^ residue exerts different structural features in Vav1 and Vav3.

Given the differential effect induced by the attachment of the H-Ras membrane anchoring signal to the Vav1^K587Q^ (rescue of activity) and Vav1^K716Q^ (no rescue) mutants, we decided to investigate whether the enforced membrane localization of Vav1 could restore normal NFAT activity levels in the case of the mutant Vav1^K222Q+K252Q^ protein (Figure 3A). We found that the Vav1^K222Q+K252Q+CAAX^ version induces levels of NFAT activity similar to those elicited by the control Vav1^CAAX^ protein (Figure S2D,E). These results indicate that the regulation mediated by the Lys^716^ must be mechanistically different from the mode of action of the acetylation sites located in the Vav1 DH and KRR regions.

### 3.7. Cooperativity of DH and SH2 Acetylation Sites on Vav1 Signaling Output

**Figure 6:**
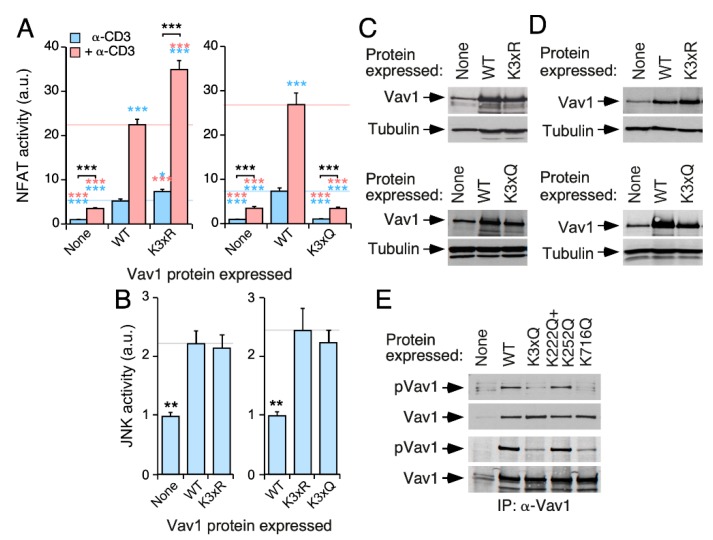
The acetylation sites located in the DH and SH2 domains cooperate in the inhibition of Vav1-mediated NFAT stimulation. (**A**,**B**) NFAT (A) and JNK (B) activation by indicated Vav1 proteins in nonstimulated (**A**,**B**) and TCR-stimulated (A) Jurkat cells. Data and *P* values are depicted as in Figure 3E (*n* = 3 independent experiments, each performed in triplicate). (**C**,**D**) Representative immunoblots showing levels of ectopic Vav1 proteins in the transfected cells used in panels A and B, respectively. Endogenous tubulin α was used as loading control. **(E)** Tyrosine phosphorylation levels of indicated Vav1 proteins immunoprecipitated from nonstimulated COS1 cells (top panel). The bottom panel shows the amount of Vav1 immunoprecipitated in each sample (*n* = 3 independent experiments). *, *p* < 0.05; **, *p* < 0.01; ***, *p* < 0.001.

To assess the consequences of the effect of the concurrent elimination of the acetylation Lys^222^, Lys^252^, and Lys^716^ sites on Vav1 activity, we generated versions of the full-length protein bearing triple Lys to Arg (K3xR) and Lys to Gln (K3xQ) mutations in those sites. We found that the ectopically expressed Vav1^K3xR^ mutant displays higher levels of NFAT activity in both nonstimulated and stimulated cells when compared with the wild-type counterpart (Figure 6A, left panel). Conversely, the Vav1^K3xQ^ mutant exhibited a total lack of NFAT activity in the same assays (Figure 6A, right panel). By contrast, we found no statistically significant effects of any of those mutations in Vav1-mediated JNK activity (Figure 6B). Western blot experiments confirmed the proper expression of the ectopic proteins used in these experiments (Figure 6C,D). Further experiments indicated that the K716Q mutation, but not the compound K222Q+K252Q mutation, affects the basal tyrosine phosphorylation state of full-length Vav1 found in nonstimulated Jurkat (Figure 6E, top panels) and COS1 (Figure 6E, bottom panels) cells. Taken together, these results confirm that the acetylation of these three acetylation sites leads to the negative regulation of the Vav1 signaling branch that favors NFAT stimulation. Given that there is not an additive effect of the two mutant subsets on Vav1 phosphorylation levels, these results further indicate that the mode of action of the DH and SH2 acetylation sites is mechanistically different.

### 3.8. Characterization of the Acetylation Lys^782^ Site Present in the Vav1 CSH3 Domain

**Figure 7:**
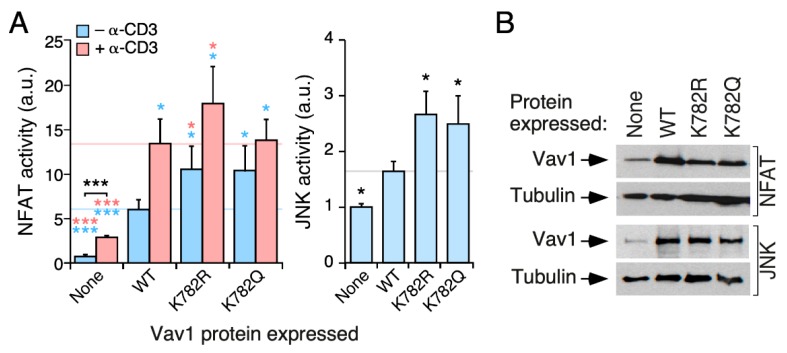
Acetylation- and deacetylation-mimicking mutations targeting the Lys^782^ residue promote the stimulation of Vav1 downstream signaling in the absence of TCR stimulation. (**A**) Activation of NFAT (left panel) and JNK (right panel) by indicated Vav1 proteins in nonstimulated and TCR-stimulated Jurkat cells. Data represent the mean ± SEM. Statistical values were obtained using the Wilcoxon matched-pairs signed rank test. Blue and salmon asterisks indicate the significance level compared with nonstimulated and TCR-stimulated Vav1^WT^-expressing cells, respectively. *n* = 3 (NFAT assays) and 4 (JNK assays) independent experiments, each performed in triplicate. (**B**) Representative immunoblots showing the abundance of the ectopic Vav1 proteins and endogenous tubulin α in the experiments shown in A. *, *p* < 0.05; ***, *p* < 0.001.

Finally, we analyzed the potential contribution of the Lys^782^ residue to the biological activity of Vav1. Although relatively poorly conserved from a phylogenetic point of view (Figure 2B and Figure S1), this is the second most acetylated site (22% of the total protein) according to our mass spectrometry analyses (Figure 2B, bottom panel). In this case, we found that the K782R and K782Q mutations promote the same stimulatory effect on the biological activity of full-length Vav1 when tested both in NFAT and JNK assays in Jurkat cells (Figure 7A,B). Given that Lys^782^ is localized in the vicinity of one of the interfaces of the CSH3 domain that contributes to the intramolecular inhibition of the protein [21], it is likely that the upregulated activity of Vav1^K782R^ and Vav1^K782Q^ proteins is owing to the release of the CSH3-mediated intramolecular inhibition of the protein (see Discussion).

## 4. Discussion

In this work, we have shown that Vav1 becomes acetylated on multiple lysine residues in a cell stimulation- and a SH2-dependent manner. In line with studies with other proteins [2,3], we have observed that the majority of the identified acetylated sites are detected at low stoichiometry under basal conditions (Figure 2B). The exception are three residues (Lys^335^, Lys^716^, and Lys^782^) that display much higher acetylation rates (10%–35% of the total protein) (Figure 2B). It is likely that such levels will become further elevated in stimulated cells, which show 2–3-fold higher levels of Vav1 acetylation according to our immunoblot analyses (Figure 1B,C and Table S2).

**Figure 8:**
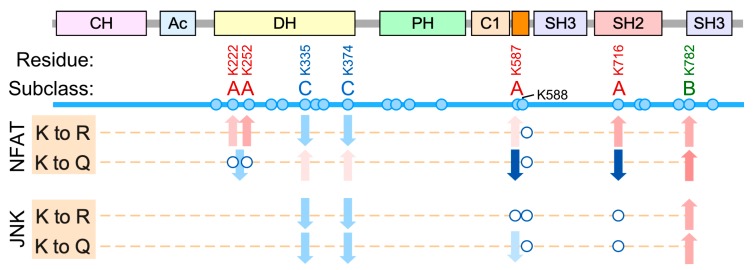
Summary of the results obtained in our work with the indicated deacetylation- and acetylation-mimetic Vav1 mutants. The residues selected for our study are shown on top. Subclass A, B, and C sites are shown in red, green, and blue, respectively. Sites with no detectable effect on Vav1 activity are shown in black. The biological assays and mutants used are indicated on the left. Red and blue arrows indicate increased and decreased stimulation of the indicated pathway, respectively, when compared to the activity of Vav1^WT^. Empty circles indicate no statistically significant differences in activity when compared to Vav1^WT^. When using a double mutant protein, the arrow is placed between the two circles.

The acetylation sites characterized in this study can be classified in three subclasses according to the impact induced on Vav1 downstream signaling (Figure 8). *Subclass A:* The acetylation of the sites belonging to this subclass leads to the specific downmodulation of Vav1-mediated activation of NFAT while it preserves the stimulation of the Rac1-JNK axis. Unlike the rest of residues interrogated in this study, we have observed that the acetylation- and deacetylation-mimicking mutations of residues of subclass A elicit different effects on the biological activity of Vav1 (Figure 8). This suggests that they constitute bonafide regulatory acetylation sites. This subgroup is composed by residues located in noncatalytic areas of the Vav1 DH domain (Lys^222^, Lys^252^), the KRR (Lys^587^), and the SH2 (Lys^716^) domains (Figure 8).

The acetylation sites of this subclass can be further subdivided into two different subsets according to their potential regulatory roles. The function of one those subsets, which includes the lysine residues located both in the KRR (Lys^587^) and the DH domain (Lys^222^, Lys^252^), seems to be associated with the regulation of the stability of the protein at the plasma membrane. Consistent with this, the inclusion of the C-terminal membrane anchoring signal restores normal signaling levels in Vav1 proteins that bear acetylation-mimetic mutations in those sites. Therefore, the regulatory role of these acetylation sites is similar to the recently described Vav1 C1-KRR phosphatidylinositol 5-phosphate binding motif [25]. Lys^587^ is probably the most relevant of those sites as judged by the dire impact that its acetylation-mimetic mutation induces on the stimulation levels of the Vav1-NFAT pathway (Figure 4A). By contrast, the mechanism of action of the remaining acetylation site (Lys^716^) is plasma membrane-independent. Our data also indicate that the role of this site is not related with the regulation of the optimal interaction of Vav1 with upstream tyrosine kinases, at least under cell stimulation conditions (Figure 5E). Although the mechanism of action of this regulatory site remains unknown, we speculate that it might be related with the maintenance of a conformation of Vav1 compatible with the optimal engagement of the NFAT pathway. We have observed similar conformation-associated effects when analyzing Vav1 proteins with mutations in specific regulatory phosphosites [21,32]. Regardless of its specific role, the Lys^716^ residue is probably one of the most relevant acetylation sites according to both stoichiometric (Figure 2B) and functional impact (Figure 5) criteria.

*Subclass B.* This functional group only contains Lys^782^, the second most acetylated Vav1 residue according to our mass spectrometric analyses (Figure 2B). The functional role of this residue is totally opposite to the rest of acetylation sites characterized in this work, since it favors the stimulation of both the Rac1-JNK and the phospholipase Cγ^−^NFAT pathways. This lysine residue is located in one of the surfaces of the CSH3 that contributes to the intramolecular inhibition of nonphosphorylated Vav1. Thus, the acetylation on this site would probably trigger a stimulation effect similar to that found upon phosphorylation of the Tyr^836^ residue also present in the CSH3 (Figure 1A) [21]. The observation of positive and negative actions of the acetylation of different lysine residues on the same protein have been described before [2]. Therefore, the detection of both subclass-A and subclass-B sites on Vav1 is not functionally incongruent if such sites are segregated in either different subcellular pools or activation time windows of the protein. However, given that the Lys^782^ to Arg and the Lys^782^ to Gln mutations elicit the same functional effect on Vav1 proteins (Figure 8), we cannot rule out the possibility that such effects could derive from the elimination of key side chains present in the replaced amino acid rather than from the Nε-acetylation step itself.

*Subclass C.* This group includes two lysine residues located in the catalytic site of the DH domain (Lys^335^ and Lys^374^). The effect of both the deacetylation- and acetylation-mimicking mutations tested for those two sites is the reduction, although not full abolition, of the Rac1-JNK axis (Figure 3 and Figure 8). In addition, they elicit very mild activation and inactivation effects on the NFAT pathway when using the acetylation- and deacetylation-mimicking mutants, respectively (Figure 3 and Figure 8). The similar effect of the Lys to Arg/Gln mutations (and even the Lys to Ala substitutions, data not shown) raises concerns on whether the dysregulation of the biological activity of Vav1 elicited by these mutations is due to acetylation-mimetic effects or to the removal of amino acid side chains that are critical for the optimal stabilization of the Vav1 DH–Rac1 interface. Regardless of this concern, these putative acetylation sites are likely less relevant than the rest of lysine residues interrogated in our study given that they are detected at very low stoichiometry levels and have not been consistently found in independent proteomics determinations (Figure 2B).

Vav1 has been primarily involved in the positive and negative selection of thymocytes as well as in the effector functions of mature T cells [6,7,17,52,53]. Based on previous evidence from catalytic-deficient *Vav1* knock-in mice, we surmise that the acetylation of the sites belonging to the functional subclass A would specifically impair previously described phospholipase Cγ- (e.g., generation of Ca^2+^ fluxes, activation of ERK and 3-phosphoinositide-dependent protein kinase-1) and NFAT-dependent (e.g., cytokine production, proliferation) responses in mature T cells. By contrast, they would preserve other functions directly associated with the catalysis-dependent pathways such as the stimulation of Rac1 itself, of the actin polymerization, of the Akt and integrin pathways, and the expression of NFAT-independent gene expression programs. The effect of the acetylation of the lysine residues that have been assigned to the subclasses B and C is more difficult to forecast given that, for example, variations in the strength of the catalytic output of the protein can elicit antagonistic effects during the positive and negative selection of immature T cells. In the case of mature T cells, we surmise that such acetylation steps would result in either the enhancement (subclass-B) or reduction (subclass-C) of the catalysis-dependent responses of Vav1 described above. Ultimately, the final outcome of these acetylation steps will be highly dependent on both the amount and functional significance of the pool of Vav1 molecules that undergoes this posttranslational modification during the stimulation cycle of both immature and mature T lymphocytes.

Our study indicates that the acetylation residues of Vav1 show low levels of phylogenetic (Figure S1) and functional (Figure S2) conservation with Vav family paralogs. This Vav1-specitity is a feature shared with the recently described C1-KRR-regulatory layer that also contributes to modulate the Vav1-mediated activation of NFAT [25]. Thus, unlike the case of the phosphorylation-dependent regulation [5], these two new regulatory layers seem to have been developed late during the evolution of the Vav family probably to fulfill Vav1- and lymphocyte-specific signaling requirements. It is worth noting that our data do not exclude the possibility that other Vav family members could be regulated by lysine acetylation as well. What they do indicate is that if that were the case, this type of regulation would involve subsets of lysine residues different from those reported here for Vav1.

## Supplementary Materials

The following are available online at https://www.mdpi.com/2073-4409/9/3/609/s1, Figure S1: Phylogenetic conservation of the indicated Vav1 acetylation sites, Figure S2: The defects associated with the acetylation of Lys^716^ are Vav1-specific, Table S1: Sequence of oligonucleotides used in this study, Table S2. Quantification of the immunoblots shown in the indicated figures of this work.
