# Supplementary material for: Lysine Acetylation Reshapes the Downstream Signaling Landscape of Vav1 in Lymphocytes
Source: Cells. 2020 Mar 4;9(3):609. doi: 10.3390/cells9030609 (PMC7140538; doi:10.3390/cells9030609)
Supplement: Supplementary file 1 [file cells-09-00609-s001.pdf]

Supplemental Information for

Lysine acetylation reshapes the downstream signaling landscape of Vav1 in lymphocytes

by

Sonia Rodríguez-Fdez, Lucía F. Nevado, L. Francisco Lorenzo-Martín and Xosé R. Bustelo*

*To whom correspondence should be addressed. E-mail: xbustelo@usal.es

This PDF file includes:

(1) Supplemental Figures S1 to S2 and legends (pages 2 to 4)

(2) Supplemental Tables S1 and S2 (pages 5 to 6)

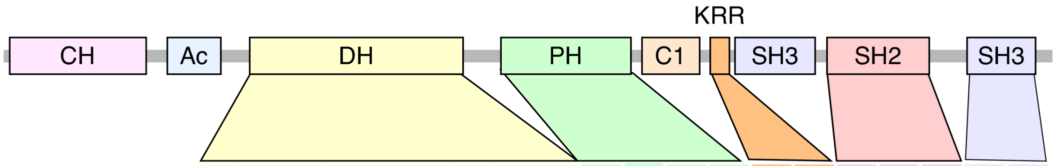

	K194	K222	K252	K292	K307	K335	K345	K353	K374	K429	K435	K444	K487	K587	K588	K716	K732	K733	K782	K815
Capsaspora owczarzaki	*	*	–	–	–	*	*	–	*	–	–	*	*	–	–	*	–	–	*	–
Salpingoeca rosetta	–	–	–	–	–	–	*	–	*	–	*	*	*	–	–	–	–	*	–	–
Amph. queenslandica	*	–	–	–	–	–	*	–	*	*	–	*	*	–	–	*	–	–	–	–
Trichoplax sp. H2	*	–	–	–	–	–	*	–	*	–	*	*	*	–	–	*	–	–	*	–
Hydra vulgaris	*	*	–	–	*	*	*	–	*	–	*	*	*	–	–	*	–	*	*	–
Clonorchis sinensis	–	–	–	–	–	*	*	–	*	*	*	–	*	–	–	–	–	–	*	–
Caenorhabditis elegans	*	–	–	*	–	*	*	–	–	*	*	*	*	–	–	*	–	–	–	–
Lottia gigantea	*	*	–	–	*	*	*	*	*	*	*	*	*	–	–	–	–	*	–	–
Helobdella robusta	*	*	*	–	–	*	*	–	–	*	*	*	*	–	–	*	–	–	*	–
Drosophila melanogaster	–	–	–	–	–	*	*	–	*	*	*	–	*	–	–	*	–	–	–	–
Daphnia magna	*	–	–	–	*	*	*	–	–	_	*	*	*	–	–	*	–	–	–	–
Branchiostoma floridae	*	–	–	–	*	*	*	*	*	*	*	*	*	–	–	*	–	*	–	–
Ciona intestinalis	*	–	–	–	–	*	*	*	*	_	*	*	*	*	*	*	–	*	–	–
Homo sapiens Vav2	*	–	–	–	*	*	–	–	*	*	*	*	*	–	–	*	–	*	–	–
Homo sapiens Vav3	–	–	–	–	*	*	*	*	*	–	*	*	*	–	*	*	–	–	*	–

FIGURE S1. Phylogenetic conservation of the indicated Vav1 acetylation sites

Black asterisks indicate the presence of a lysine residue in the primary sequence of the indicated Vav family protein that is conserved in mouse Vav1. Lysine residues identified as acetylation sites in other Vav family proteins using high-throughput proteomics analyses are indicated in red.

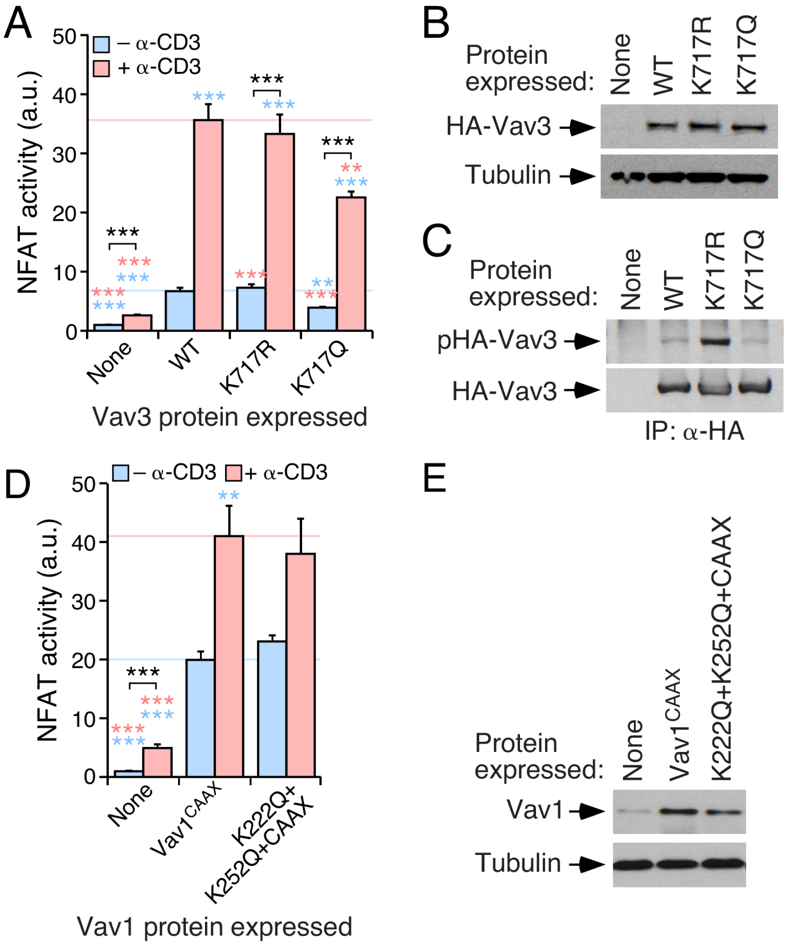

FIGURE S2. The defects associated with the acetylation of Lys^{716} are Vav1-specific

(A) Activation levels of NFAT triggered by indicated HA-tagged Vav3 proteins in nonstimulated and TCR-stimulated Jurkat cells. Data represent the mean ± SEM. Statistical values were obtained using the Mann-Whitney U test. Blue and salmon asterisks indicate the significance level compared with nonstimulated and TCR-stimulated HA-Vav3^{WT}-expressing cells, respectively. Black asterisks refer to the P values obtained between the indicated experimental pairs (in brackets). n = 3 independent experiments, each performed in triplicate.

(B) Representative immunoblots showing the abundance of the ectopic HA-Vav3 proteins and endogenous tubulin α in the experiment shown in A.

(C) Tyrosine phosphorylation levels of the indicated HA-Vav3 proteins (top) immunoprecipitated from exponentially growing COS1 cells. Similar results were obtained in 3 independent experiments.

(D) Activation levels of NFAT induced by the indicated Vav1 proteins in in nonstimulated and TCR-stimulated Jurkat cells. Data represent the mean ± SEM. Statistical values were obtained using the Mann-Whitney U test. Blue and salmon asterisks indicate the significance level compared with nonstimulated and TCR-stimulated Vav1^{CAAX}-expressing cells, respectively (n = 3 independent experiments, each performed in triplicate).

(E) Representative immunoblots showing the abundance of the ectopic Vav1 proteins and endogenous tubulin α in the experiments shown in D.

TABLE S1. Sequence of oligonucleotides used in this study

Mutant	DNA sequence of primer	
Vav1^{K222R}	F*	5'– CAGCAGCACTTCATGAGGCCTCTGCAGCTATTC –3'
	R	5'– GAATCGCTGCAGAGGCCTCATGAAGTGCTGCTG–3'
Vav1^{K222Q}	F	5'– CAGCACTTCATGCAGCCTCTGCAGC –3'
	R	5'– GCTGCAGAGGCTGCATGAAGTGCTG –3'
Vav1^{K252R}	F	5'– GCATACCCACTTCTTAAGGGAACTGAAGGATGCCC –3'
	R	5'– GGGCATCCTTCAGTTCCCTTAAGAAGTGGGTATGC–3'
Vav1^{K252Q}	F	5'– GCATACCCACTTCTTACAGGAACTGAAGGATGC –3'
	R	5'– GCATCCTTCAGTTCCTGTAAGAAGTGGGTATGC –3'
Vav1^{K335R}	F	5'– ATGCAGCGGGTGCTGAGGTACCACCTCCTTCTC –3'
	R	5'– GAGAAGGAGGTGGTACCTCAGCACCCGCTGCAT–3'
Vav1^{K335Q}	F	5'– CCTATGCAGCGGGTGCTGCAGTACCACCTCCTTCTCC –3'
	R	5'– GGAGAAGGAGGTGGTACTGCAGCACCCGCTGCATAGG –3'
Vav1^{K374R}	F	5'– TGCGTGAACGAGGTCAGGAGGGACAATGAAACC–3'
	R	5'– GGTTTCATTGTCCCTCCTGACCTCGTTCACGCA –3'
Vav1^{K374Q}	F	5'– GTGCGTGAACGAGGTCCAGAGGGACAATGAAAC –3'
	R	5'– GTTTCATTGTCCCTCTGGACCTCGTTCACGCAC –3'
Vav1^{K587R}	F	5'– GGCCCAGGACAGGAAAAGGAATG –3'
	R	5'– CATTCCTTTTCCTGTCCTGGGCC –3'
Vav1^{K587Q}	F	5'– GGGCCCAGGACCAGAAAAGGAATG –3'
	R	5'– CATTCCTTTTCTGGTCCTGGGCCC –3'
Vav1^{K588R}	F	5'– GGCCCAGGACAAGAGAAGGAATGAATTGG –3'
	R	5'– CCAATTCATTCCTTCTCTTGTCCTGGGCC –3'
Vav1^{K588Q}	F	5'– GGGCCCAGGACAAGCAAAGGAATGAATTG –3'
	R	5'– CAATTCATTCCTTTGCTTGTCCTGGGCCC –3'
Vav1^{K716R}	F	5'– CAGCATTAAGTATAACGTGGAGGTCAGGCATATTAAAATCATGACGTCAGA –3'
	R	5'– CCTCTGACGTCATGATTTTAATATGCCTGACCTCCACGTTATACTTAATGC–3'
Vav1^{K716Q}	F	5'– CATTAAGTATAACGTGGAGGTCCAGCATATTAAAATCATGACGTC –3'
	R	5'– GACGTCATGATTTTAATATGCTGGACCTCCACGTTATACTTAATG –3'
Vav1^{K782R}	F	5'– CAGCTGGAAGCACCAGGTATTTTGGCACTGC –3'
	R	5'– GCAGTGCCAAAATACCTGGTGCTTCCAGCTG –3'
Vav1^{K782Q}	F	5'– CCAGCTGGAAGCACCCAGTATTTTGGCACTG –3'
	R	5'– CAGTGCCAAAATACTGGGTGCTTCCAGCTGG –3'
Vav3^{K717R}	F	5'– GTACAATAATGAAGCAAGGCACATCAAGATTTTAAC –3'
	R	5'– GTTAAAATCTTGATGTGCCTTGCTTCATTATTGTAC –3'
Vav3^{K717Q}	F	5'– CAATAATGAAGCACAGCACATCAAG –3'
	R	5'– CTTGATGTGCTGTGCTTCATTATTG –3'

*F, forward primer; R, reverse primer. Nucleotides that have been replaced in the WT *Vav1* coding sequence to incorporate the indicated point mutations are shown in red.

TABLE S2. Quantification of the immunoblots shown in the indicated figures of this work

Figure	Time (min)	Protein expressed	Lysine acetylation[a] mean ± SEM (a.u.)	Tyrosine phosphorylation mean ± SEM (a.u.)
1B	**0**	None	**1.00±0.00**	**1.00±0.00**
	2	None	2.78±0.30**,[b]	4.28±0.44**
	5	None	2.65±0.25**	4.44±0.54**
	10	None	2.14±0.31*	4.16±0.64*
	15	None	1.55±0.17	4.85±0.84*
	30	None	1.00±0.07	3.41±0.81
1C	0	None	–	–
	**0**	**$Vav1^{WT}$**	**1.00±0.00**	**1.00±0.00**
	5	$Vav1^{WT}$	2.16±0.020**	2.16±0.09***
	10	$Vav1^{WT}$	2.08±0.16**	2.24±0.07***
	15	$Vav1^{WT}$	2.07±0.42**	2.35±0.22**
	30	$Vav1^{WT}$	1.61±0.26	2.03±0.08**
1D		None	–	
		**$Vav1^{WT}$**	**1.00±0.00**	
		$Vav1^{P651L}$	0.80±0.18	
		$Vav1^{G691V}$	0.53±0.08**	
		$Vav1^{P833L}$	1.61±0.56	

[a]Values were quantitated and normalized as indicated in Methods (section 2.5). [b]Statistical significance was calculated using 2-way ANOVA (Fig. 1B and C) or Mann-Whitney U test (Fig. 1D) relative to the values obtained with the indicated control (shown in bold, which was given an arbitrary value of 1 in each case). *, $P < 0.05$; **, $P < 0.01$; ***, $P < 0.001$.
